# Occurrence, Ecological Risk, and Source Apportionment of Antibiotics in Surface Water and Sediment of Yellow River Delta

**DOI:** 10.3390/toxics14070552

**Published:** 2026-06-25

**Authors:** Jinghao Wang, Shaohua Zhang, Yaoshen Fan, Feihe Kong, Renjie Huang, Shentang Dou

**Affiliations:** 1 Yellow River Institute of Hydraulic Research, Yellow River Conservancy Commission, Zhengzhou 450003, China; wang2022hky@126.com (J.W.); shaohuazhang1@163.com (S.Z.); fysmyself@126.com (Y.F.); feihek@stumail.nwu.edu.cn (F.K.); 13298313336@163.com (R.H.); 2 Yellow River Estuary Eco-Hydrology Field Research Station, Key Laboratory of Yellow River, Ministry of Water Resources, Zhengzhou 450003, China

**Keywords:** antibiotics, spatial–temporal distribution, environmental risk assessment, source apportionment

## Abstract

The Yellow River Delta (YRD), a crucial ecotone, is becoming increasingly polluted by antibiotics, posing serious threats to aquatic ecosystems and human health. In this study, comprehensive investigations were conducted to explore the regional distribution, environmental risks, and source apportionment of antibiotics, with the aim of facilitating precise management and control of antibiotic pollution. The results show that the surge in runoff during the water–sediment regulation period (June and August) of the Yellow River drove a sharp rise in antibiotic concentrations in the surface water, peaking at 135.0 ng/L, whereas antibiotics were rarely detected in the sediments after multiple rounds of intense hydraulic scouring (0.2~12.6 ng/g in October). Furthermore, seven antibiotics (sulfadiazine, sulfamethoxazole, flumequine, ofloxacin, tetracycline, doxycycline, and lincomycin) in surface water and six antibiotics (norfloxacin, enrofloxacin, ofloxacin, doxycycline, oxytetracycline, and florfenicol) in sediments were identified as representative compounds according to the antibiotic pollution profiles. Environmental risk assessment coupled with spatial autocorrelation analysis revealed that sulfamethoxazole generally posed medium to high risk (0.12~1.27) in surface water. Sediments posed more serious ecological risks, with universally high risk levels (ranging from 1.11 to 280.00). More importantly, in both surface water and sediment, four core antibiotic sources—namely, human sewage, livestock farming, agricultural and aquaculture inputs, and hydrodynamic-driven resuspension processes—were consistently identified through the Positive Matrix Factorization model and Kriging interpolation. These findings provide crucial insights for establishing targeted antibiotic pollution control strategies in the YRD and advance the understanding of antibiotic fate in sediment-laden rivers.

## 1. Introduction

Due to their high usage levels and limited removal during wastewater treatment, antibiotics inevitably flow into and converge in river systems. Their environmental persistence and bioaccumulation can induce toxicity at multiple biological levels, affecting genes, cells, populations, ecosystems, etc. [1,2,3,4,5]. As is well documented, antibiotic exposure of surface water in Peñas Blancas River could selectively shape microbial assemblages to cause significant compositional shifts, with the enrichment of *Aurantimicrobium* and *Sphingomonas* and the decline of *Bdellovibrio* and *Methylomonas* [6]. Enrofloxacin (ENR) and oxytetracycline (OTC) in sediments of the Douro River Estuary have decreased the richness and diversity of the microbial community and altered the dominant phyla [7]. Notably, changes in functional populations directly disturb biogeochemical cycling processes. Chen et al. found that exposure to sulfamethoxazole (SMX) in Yangtze Estuary sediments with near in situ concentrations (0.3–56.8 ng/L) could inhibit nitrogen transformation functional genes, including the ammonia monooxygenase gene (*amoA*, a marker for ammonia-oxidizing bacteria (AOB)), encoding subunit A of ammonia monooxygenase; the nitrate reductase gene (*narG*), encoding the catalytic molybdenum-cofactor-containing subunit of membrane-associated nitrate reductase; the nitrite reductases gene (*nirS*), encoding cytochrome cd1; the nitrous oxide reductase gene (*nosZ*); and the anammox 16S rRNA gene, thereby decreasing nitrogen removal rates [8]. Similarly, Yamamura et al. proved that chloramphenicol (CP) could strongly inhibit aerobic As(III) oxidation by influencing As(III) oxidase genes and *Proteobacteria*-related 16S rRNA [9]. Moreover, the persistent exposure of microbes to antibiotics accelerated the production and enrichment of antibiotic resistance genes (ARGs), which were also proved to influence the carbon, nitrogen, and/or sulfur cycles [10,11]. Furthermore, antibiotics can diffuse from the environmental medium into organisms. Quinolones in mollusks of the Bohai Sea of China reached astounding levels of 0.71–1575.10 ng/g [12]. Similarly, in terrestrial food products, studies from Egypt and Bangladesh reported that tylosin (TYL), CP, OTC, and ciprofloxacin (CIP) have also been frequently detected in liver, kidney, and muscle tissues of broiler chickens [13]. However, antibiotics remain unregulated, with no limits imposed under existing conventions and standards and no detailed framework for investigating and governing their levels. This institutional deficiency has probably facilitated severe pollution incidents or ecological tragedies [14]. Therefore, detecting the occurrence of antibiotics and identifying those that pose the high risk for priority monitoring are imperative.

Although antibiotic occurrence has been frequently studied in rivers [15,16,17], lakes [18,19,20], groundwater [21,22], and the ocean [23,24], estuarine deltas, as critical interfaces connecting freshwater and marine systems, have been rarely explored. As ecologically sensitive interface areas, estuarine deltas are simultaneously influenced by the hydrological, geomorphological, and physicochemical characteristics of terrene, freshwater, and marine, which creates a unique biodiversity [25,26]. With the rapid development of urbanization, industrialization, and aquaculture, runoff and anthropogenic activities have introduced massive pollutant loads into these ecosystems, inevitably making them the transport and storage areas of antibiotics [27,28,29].

Notably, estuarine deltas generally exhibit conventional hydrodynamic features, with marked seasonal variations and frequent material exchanges due to their special geographical locations [30]. However, the YRD is further shaped by regular anthropogenic flow regulation, specifically a unique water–sediment regulation scheme aimed at removing sand and reducing silt in response to the delta’s exceptionally high suspended sediment load. This regulation generates episodic flood pulses lasting several days, during which flow velocity and bed shear stress increase dramatically, triggering intense sediment resuspension and pronounced hydrodynamic sorting of sediment particles [31,32,33]. Consequently, antibiotics that have accumulated in bottom sediments can be remobilized into the water column, converting the delta from a temporary sink into a secondary source. Large-scale resuspension and settling of particles, combined with the salinity gradient in the estuary, further complicate the antibiotic distribution and behavior. However, studies on antibiotics in the YRD are lacking; most studies have merely described the antibiotic residues in water bodies or sediments, ignoring the sources of the antibiotics. Addressing this gap can be expected to not only advance our understanding of antibiotic fate in this extreme setting but also provide a reference for assessing antibiotic behavior in other large rivers where either high sediment loads or anthropogenic flow regulation plays a dominant role.

Therefore, in this study, antibiotics widely detected in surface water and sediments were selected to (i) analyze their spatial–temporal distribution in the YRD and identify species representative of the region, (ii) assess the environmental risk and establish the priority control list, and (iii) conduct source apportionment to support antibiotic management and control.

## 2. Materials and Methods

### 2.1. Research Area and Sample Collection

Surface water and sediment samples from the YRD (Dongying, Shandong Province, China) were collected in April, June, August, and October of 2024 (Figure S1). Considering the differences in ecological environments, the selected sampling sites were in the Yellow River Estuary (YRE, Y1–Y7), Old Yellow River (OYR, O1–O4), and the mariculture area (MA, M1–M2), the distribution of which is shown in Figure S2.

All samples were obtained in triplicate. Specifically, water samples were collected at a depth of 0.30 m below the water surface using a transparent organic glass water sampler. The collected samples were mixed thoroughly and stored in amber glass bottles under refrigeration. This water sampler conformed to Chinese water quality—guidance on sampling techniques (HJ 494-2009). It was an open-ended, transparent, organic glass cylinder equipped with a scale, a thermometer, a bottom weight, and a lower-side rubber tube. Both ends had paired semicircular flaps that opened upward during descent and closed automatically upon ascent due to water pressure and gravity.

Sediment samples were taken using a stainless-steel grab sampler and stored in double-layered polyethylene plastic bags. Subsequently, the sediment samples were cleaned of impurities (e.g., gravel and plant roots), freeze-dried, ground, and sieved. It should be noted that sediment sampling was suspended in June and August due to high river discharge, precluding navigation (>2000 m^3^/s).

### 2.2. Chemicals and Reagents

The standard forms of all target antibiotics/antifungal drugs were purchased from Anpu Experimental Technology Co., Ltd. (Shanghai, China), including 17 sulfonamides (SAs) and their corresponding antibacterial enhancers, 16 quinolones (QNs), 6 macrolides (MLs), 4 tetracyclines (TCs), 3 chloramphenicols (CPs), 2 imidazoles (MIs), and 1 lincosamide (LM) (Table S1). HPLC-grade acetonitrile and methanol were purchased from Tedia Company, Inc. (Fairfield, OH, USA). Other chemicals were of analytical grade. Ultrapure water was supplied by a Milli-Q system (Millipore, Billerica, MA, USA).

### 2.3. Sample Extraction

The samples were extracted using the following procedures, as described in a previous study [34].

For the water samples, suspended particulates were removed by vacuum filtration through 0.45 μm glass fiber filter membranes. The obtained filtrates were mixed with Na_2_EDTA (0.25 g), and H_2_SO_4_ (0.22 mol/L) was added dropwise to adjust the pH to 3. Then, the treated water samples were input into preconditioned HLB solid-phase extraction (SPE) cartridges (200 mg/6 mL, Anpu Experimental Technology Co., Ltd. (Shanghai, China)), which were sequentially activated with methanol (6 mL) and ultrapure water (6 mL). The loaded HLB cartridges were rinsed with ultrapure water and dried for 2 h; subsequently, the target antibiotics/antifungal drugs enriched in the SPE cartridges were successively eluted with methanol and methanol containing 5% aqueous ammonia. The collected eluents were evaporated under a nitrogen flow to 0.2 mL and re-constituted in an acetonitrile/0.1% formic acid solution (1/1, *v*/*v*). Finally, the extracts were filtered through 0.22 µm PTFE fiber filters for subsequent analysis.

For sediment samples, freeze-dried sediment (2.0 g) was first mixed with 8 mL of a solution containing 50% Mg(NO_3_)_2_ and 10% NH_4_OH (96:4, *v*/*v*). The mixture was vortexed (3 min), sonicated (15 min), and centrifuged (5000 rpm, 5 min) in sequence. The collected supernatant was labeled A. The remaining precipitate was then extracted with 20 mL of ultrapure water by vortexing (3 min) and centrifugation (5000 rpm, 5 min), and the supernatant obtained in this process was labeled B. Finally, the residue was added to a mixture of Na_2_EDTA (0.2 g), phosphate-buffered saline (5 mL, 220 mM, pH = 2.5), and acetonitrile (5 mL) and then vortexed (3 min), sonicated (15 min), and centrifuged (5000 rpm, 10 min). This step was repeated three times, and the combined supernatants were labeled C. All supernatants (A, B, and C) were transferred into a flask and diluted to 500 mL with ultrapure water, and the pH was adjusted to 3.0 (Figure S3). The diluted solution was enriched using the same procedure as that described for the water samples.

### 2.4. Antibiotic Detection

Ultra-high-performance liquid chromatography–tandem mass spectrometry (UHPLC-MS/MS) was used to identify and quantify antibiotic residues in water and sediment samples. In detail, 5 μL of extracted samples (see Section 2.3) were separated by an ACQUITY BEH C18 column (2.1 mm × 100 mm, 1.7 μm) with a flow rate of 0.4 mL/min. The mobile phase was set to 0.1% formic acid aqueous solution (A) and methanol (B) to conduct gradient elution (0–0.25 min, 90% A and 10% B; 0.25–4.50 min, 90% A to 60% A; 4.50–6.00 min, 60% A to 5% A; 6.00–8.00 min, 5% A and 95% B; 8.00–10.00 min, 90% A and 10% B). Multiple reaction monitoring in ESI+ (positive) and ESI− (negative) modes of the Xevo TQ system was employed to quantify the target antibiotics. The measurement conditions were set as previously described [34,35].

### 2.5. Quality Assurance and Quality Control

The limits of detection (LODs), limits of quantification (LOQs), average recovery, linearity, and precision were determined to validate the reliability of the methods in every batch of samples. LODs and LOQs were defined as 3 and 10 times the signal-to-noise ratio (S/N), respectively. Notably, unspiked samples were prepared to serve as blank controls and treated using identical analytical procedures to detect any contamination caused by human operator error and interference from environmental factors. Simultaneously, solvent blanks and standards (ng/L) were analyzed to assess the stability of system performance. The method validation parameters for target antibiotics are shown in Table S2.

### 2.6. Risk Assessment

The risk quotient (RQ) method was used to conduct an ecological risk assessment of the representative antibiotics detected in the YRD. The RQ formula used is presented in the following equation. The calculated RQ values were used to classify the ecological risk of individual antibiotics as high (RQ ≥ 1.0), medium (0.1 ≤ RQ < 1.0), low (0.01 ≤ RQ < 0.1), and insignificant (RQ < 0.01).

(1) $${\mathrm{RQ}}_{\mathrm{water}/\mathrm{sed}}=\frac{{\mathrm{MEC}}_{\mathrm{water}/\mathrm{sed}}}{{\mathrm{PNEC}}_{\mathrm{water}/\mathrm{sed}}}$$

(2) $${\mathrm{PNEC}}_{\mathrm{water}}=\frac{\mathrm{Min}{\left({\mathrm{EC}}_{50\mathrm{Algae}},\;{\mathrm{EC}}_{50\mathrm{Crustacean}},{\mathrm{EC}}_{50\mathrm{Fish}}\right)}_{i}}{\mathrm{AF}}$$

(3) $${\mathrm{PNEC}}_{\mathrm{sed}}=\frac{K_{\mathrm{susp}-\mathrm{water}}}{{\mathrm{RHO}}_{\mathrm{susp}}}\times {\mathrm{PNEC}}_{\mathrm{water}}$$

(4) $$K_{\mathrm{susp}-\mathrm{water}}=F_{\mathrm{water}-\mathrm{susp}}+F_{\mathrm{solid}-\mathrm{susp}}\times \frac{{\mathrm{Kp}}_{\mathrm{susp}}}{1000}{\mathrm{RHO}}_{\mathrm{solid}}$$

(5) $${\mathrm{Kp}}_{\mathrm{susp}}=0.1\;\times \;K_{\mathrm{oc}}$$

(6) $$\log K_{\mathrm{oc}}=0.623\;\log K_{\mathrm{OW}}\;+\;0.873$$

where ${\mathrm{MEC}}_{\mathrm{water}/\mathrm{sed}}$ and ${\mathrm{PNEC}}_{\mathrm{water}/\mathrm{sed}}$ are the measured environmental concentration and the predicted no-effect concentration of target antibiotics in the corresponding medium (ng/L in surface water or ng/g in sediment), respectively. *EC_50 Algae_*, *EC_50 Crustacean_*, and *EC_50 Fish_* represent the half-maximal effective concentrations of individual antibiotics for algae, crustaceans, and fish, which can be obtained from the Ecotoxicology Database (ECOTOX) of the United States or predicted using the ECOSARv.2.2 software (Tables S3 and S4). According to the Technical Guidance Document on Risk Assessment, AF, *RHO_susp_*, *RHO_soild_*, *F_water-susp_*, and *F_solid-susp_* are the evaluation factor, bulk density of suspended matter, bulk density of wet soil, volume fraction of water in suspended matter, and volume fraction of solids in suspended matter, with recommended values of 1000, 1150 kg·m^−3^, 2500 kg·m^−3^, 0.9, and 0.1, respectively. *Kp_susp_*, *K_oc_*, and *K_ow_* are the solid–water partition coefficient in suspended matter, the organic carbon–water partition coefficient, and the octanol–water partition coefficient, respectively.

The cumulative risk quotient (CRQ) was also calculated in this study to evaluate the ecological risk of the representative antibiotics in the YRD.

(7) $${\mathrm{CRQ}}_{j}=\sum_{i=1}^{n}{\mathrm{RQ}}_{i}$$

To exclude the influence of the number of values and outliers [36], the weighted frequency of RQ (WFRQ) was calculated for the targeted management of antibiotics.

(8) $$\mathrm{WFRQ}=\sum_{i=1}^{4}F_{x}W_{x}$$

where *F_x_* represents the proportion of RQ values corresponding to rank level x in the total RQ number for a specific antibiotic, and x is assigned a value from 1 to 4 for high risk (RQ ≥ 1.0), medium risk (0.1 ≤ RQ < 1.0), low risk (0.01 ≤ RQ < 0.1), and insignificant risk (RQ < 0.01), respectively. *W_x_* is the corresponding weighting index; i.e., *W*_1_ is set to 1, *W*_2_ is set to 0.5, *W*_3_ is set to 0.25, and *W*_4_ is set to 0.

### 2.7. Data Analysis

ArcGIS (version 10.8) and Origin software (version 2024) were jointly used to show the geographic information of sampling points and the distribution of target antibiotics. All data were analyzed via one-way ANOVA. Statistical analysis was carried out to evaluate significant differences between experimental data (*p <* 0.05).

The Positive Matrix Factorization (PMF, version 5.0) model, developed by the United States Environmental Protection Agency (US EPA), was employed to conduct the source apportionment of representative antibiotics identified in surface water and sediment. In brief, concentration values (*Con.*) below the method detection limit (MDL) were replaced by 1/2 MDL, and the corresponding uncertainty (*Unc.*) was set to 5/6 MDL. For *Con.* above the MDL, *Unc.* was calculated based on Equation 2.9.

(9) $$Unc.=\sqrt{{(\mathrm{RSD}\;\times \;Con.)}^{2}+{\left(0.5\;\times \;\mathrm{MDL}\right)}^{2}}$$

where RSD represents relative standard deviation. The inter-day RSD was employed in this study to assess the long-term random error in the analysis of trace antibiotics in the environment.

The factor contribution values obtained from the PMF model were imported into the ArcGIS software (version 10.8), in which Kriging interpolation was used to visually identify the spatial distribution patterns of pollution sources.

## 3. Results and Discussion

### 3.1. Accumulation and Temporal–Spatial Distribution of Antibiotics

**Figure 1:**
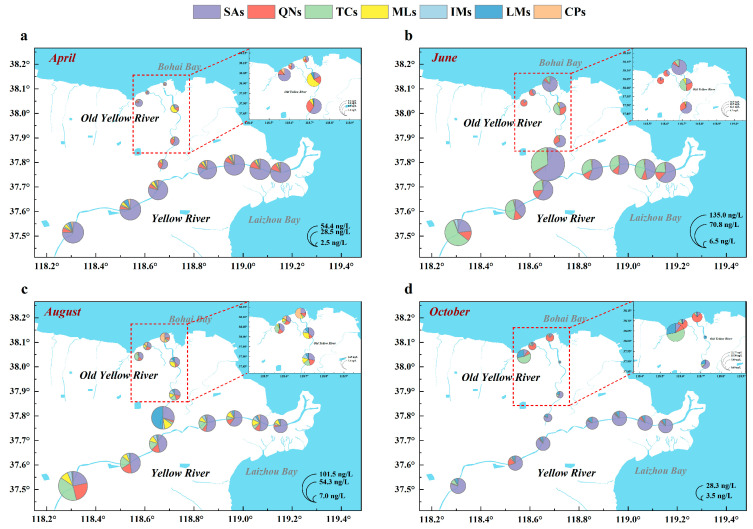
Temporal–spatial distribution of antibiotics in surface water samples collected in April (**a**), June (**b**), August (**c**), and October (**d**). The diameter of the black semicircle in the legend represents the total cumulative concentration of the target antibiotic. Map lines delineate study areas and do not necessarily depict accepted national boundaries.

In surface water, all target antibiotic classes were detected with residual concentrations at the ng/L level (Figure 1). In contrast, antifungal drugs (i.e., IMs, including ketoconazole and miconazole) showed low concentrations, so they are not further discussed in this study. The cumulative antibiotic concentrations in the YRE and OYR varied in different periods, separately ranging from 1.4 to 54.3 (April), 4.7 to 135.0 (June), 7.3 to 101.4 (August), and 0.7 to 28.3 ng/L (October). The concentrations were one order of magnitude higher in June and August than in the other months, with the differences determined to be significant. This phenomenon differs from the typical seasonal pattern, in which higher antibiotic accumulation occurs in the dry season compared to the wet season, as observed in Poyang Lake [37], Haihe River [38], and Yangtze River [3]; this divergence was mainly attributed to the unique water–sediment regulation system of the Yellow River. The sharp increase in flow discharge in the water–sediment regulation period (June and August) carries antibiotics upstream to converge in the YRD. In contrast, the cumulative concentrations in the MA were hardly influenced by water–sediment regulation and only slightly increased in October (6.7~22.8 ng/L vs. 1.5~9.1 ng/L) due to water exchange in culture ponds. Moreover, antibiotic concentrations at sample points in the YRE (17.9~101.4 ng/L) showed significantly higher accumulation levels than those in the OYR (0.7~26.9 ng/L, except for O1, near a hennery) and MA (1.5~22.8 ng/L) in all sampling periods, suggesting considerable variations in spatial distribution. In addition to the upstream pollution inflow causing the above phenomenon, Y1–Y5, surrounded by agricultural land, suffered an inevitable influx of antibiotics from animal organic fertilizer due to conventional flood irrigation, and Y6–Y7 received antibiotic input carried by exchanges between river water and seawater.

**Figure 2:**
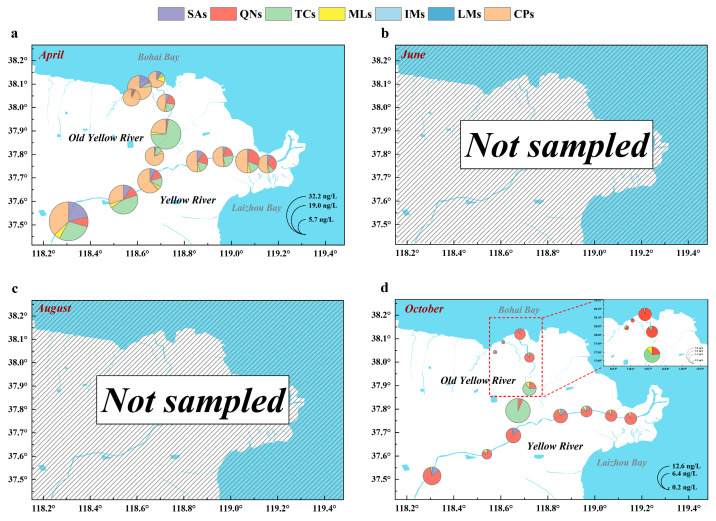
Temporal–spatial distribution of antibiotics in sediment samples collected in April (**a**) and October (**d**). Sediment sampling in June (**b**) and August (**c**) was suspended, as indicated by graphical placeholders labeled “Not sampled”. The diameter of the black semicircle in the legend represents the total cumulative concentration of the target antibiotic. Map lines delineate study areas and do not necessarily depict accepted national boundaries.

Meanwhile, all target antibiotic classes except LMs were also detected in sediment samples with residual concentrations at the ng/g level (Figure 2); the cumulative concentrations were 5.8~32.2 (April) and 0.2~12.6 ng/g (October). Enhanced hydrodynamic scour during the water regulation stage intensified selective-entrainment processes, increasing bulk-sediment grain size while transporting fine-grained fractions offshore. Since micro-organic pollutants preferentially associate with fine particles under low water flow, the predominance of coarser fractions in the sediment after multiple strong hydrodynamic scour events between April and October (Figure S1) could explain the lower accumulation level in October [32,33,39].

### 3.2. Analysis of Dominant Antibiotic Classes

The average percentages of the target antibiotic classes in three region subsets (YRE, OYR, MA) with diverse geographic features were calculated to identify the dominant pollutants.

As shown in Figure S4, the dominant antibiotics in the surface water of the YRE were almost identical in each sampling period; this notable consistency is due to the absence of tributary dilution and direct influence of upstream input. In particular, the average percentage of SAs in the YRE reached 64.8%, much higher than those of other antibiotics. TCs, QNs, and MLs accounted for 14.3%, 9.8%, and 4.4% of the total antibiotic load, respectively. The overwhelming predominance of SAs was attributed to their generally high water solubility (610~12,500 mg/L), strong hydrophilicity (log*K_ow_* = −1.22~1.68), and chemical stability, which renders them resistant to photolysis, hydrolysis, and biodegradation [35,40]. Notably, SAs were also primary contaminants of the OYR, with an average percentage of 42.6%. This similarity was mainly attributed to the simultaneous ecological water replenishment in the OYR from the YRE during the water–sediment regulation period, which strengthened the hydraulic connection between the two areas. Additionally, episodic effluent from petrochemical industry wastewater and frequent application of animal fertilizer in agricultural lands resulted in a more mixed profile in the OYR [35], with QNs (18.3%), LMs (14.5%), and MLs (9.7%). Differing from the YRE and OYR, the MA was mainly polluted by QNs (38.5%), which is consistent with their frequent use in aquaculture in the Shandong Peninsula [41]. Additionally, photolysis or biodegradation of QNs would be inhibited in the MA due to its high salinity.

In contrast to surface water, sediment had negligible levels of LMs and IMs, with average percentages less than 1%, and the dominant antibiotics were QNs (30.1%~43.9%) or TCs (10.3%~35.9%) instead of SAs (2.3%~8.4%), which could be explained by their high partition coefficients (*K_d_*) (8.13~42,500 L/kg) in our study. Additionally, QNs and TCs possess carbonyl/hydroxyl (p*K_a_*: 5~7.7), amino (p*K_a_*: 7~10), and other polar groups, leading to complexation with cations (e.g., Na^+^, Ca^2+^, Fe^3+^) and organic matter in sediments (pH: 8.08~9.34 in our study) [42]. Furthermore, the detected antibiotics markedly differed in temporal distribution. Significantly more antibiotic classes (QNs, TCs, SAs, CPs, etc.) were detected in April, especially CPs (47.6%~84.2%), which only occurred in this period. This phenomenon might reflect the fact that April was the peak period for seedling release in aquaculture and antibiotic administration for epidemic prevention in livestock.

### 3.3. Identification of Representative Antibiotics

To further identify the representative antibiotics, the detected concentrations and frequencies of the above-mentioned classes were statistically analyzed.

**Figure 3:**
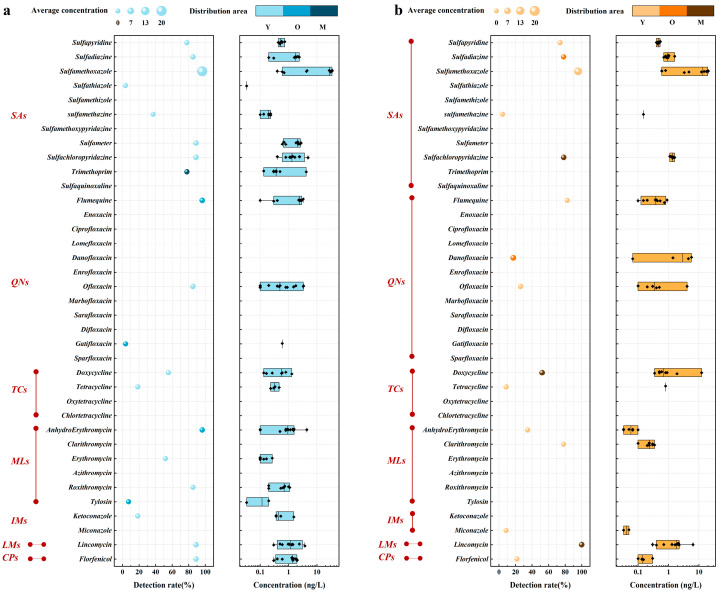
The average concentrations and detected frequencies of antibiotics in surface water ((**a**): April; (**b**): October). Y, O, and M represent YRE, OYR, and MA, respectively. The bubble color signified the region where the sampling point with the highest detected concentration is located.

Among 50 target compounds (Table S1), a total of 36 antibiotics and 1 synergist (corresponding to SAs) were detected in the four sampling campaigns of surface water. As reflected in Figure 3 and Figure S5, the antibiotic diversity and concentrations distinctly increased when the flow discharge rose sharply in the water–sediment regulation period, rather than being caused by surface runoff, as reported in other studies [43]. Thereinto, four SAs were detected with high frequencies (82.6%~100%) and high average concentrations (1.6~19.3 ng/L) more than once: sulfadiazine (SDZ), sulfamethoxazole (SMX), sulfameter (SMT), and sulfachloropyridazine (SCP). SMX and SDZ, which are generally used in combination with trimethoprim, were present at almost all sampling points, with maximum concentrations reaching 34.4 (April) and 78.4 ng/L (June), much higher than those measured in the Yellow River mainstream [28], urban surface water in Singapore [44] and Brahmaputra River in Bangladesh [45]. Flumequine (FLU) and ofloxacin (OFL), antibiotics belonging to the QN class, were persistent, with high detected frequencies (82.6%~100%). These compounds have rarely been detected in the Yellow River [28,35], let alone at such high levels: the average concentrations of FLU (0.4~4.1 ng/L) were almost comparable to those in the Yangtze River [3] and Haihe River [46]. In contrast, although TCs have been detected in the Yellow River in previous studies [28], their average concentrations in this study were observably higher, especially in June (6.4~13.6 ng/L), when the maximum detected concentrations of tetracycline (TET) and doxycycline (DOX) were as high as 36.4 and 15.9 ng/L, respectively, which were significantly lower than those in Ghana [47], the USA [48] and Canada [49], possibly reflecting intercountry variations in the regulatory control of antibiotic use. In addition, anhydroErythromycin (ETM-H_2_O), clarithromycin (CLA), and roxithromycin (ROX)—MLs that show significant positive correlations with population density—were also detected with relatively high concentrations or frequencies. The maximum concentration of lincomycin (LIM, log*K_ow_* = 0.2) reached 31.0 ng/L.

Overall, the peak concentrations of 36 detected antibiotics were mainly distributed in the YRE, followed by the OYR and MA, reflecting the levels of pollution in the three region subsets. In these regions, SMX, SDZ, FLU, OFL, TET, DOX, and LIM were regarded as representative antibiotics present in surface water.

**Figure 4:**
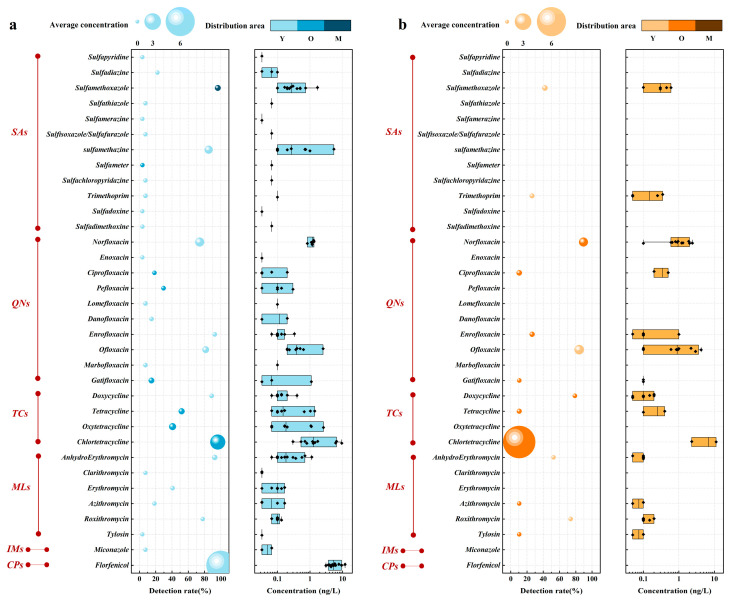
The average concentrations and detected frequencies of antibiotics in sediments ((**a**): April; (**b**): October). Y, O, and M represent YRE, OYR, and MA, respectively. The bubble color signified the region where the sampling point with the highest detected concentration is located.

Furthermore, 33 target compounds were detected in sediment samples in April, but only 14 target compounds were found in October (Figure 4).

In particular, the detected antibiotics with the higher frequencies and concentrations were classified as QNs, TCs, and CPs, consistent with the above analysis of the dominant antibiotic classes. Norfloxacin (NOR), enrofloxacin (ENR), ofloxacin (OFL), doxycycline (DOX), chlortetracycline (CTC), and FLO were the main representatives. Interestingly, their concentrations (0.05–12.0 ng/g) were significantly lower than those found in the Yangtze River [3] and Poyang Lake [37], probably due to the high suspended particle content in the YRD, which caused antibiotics to preferentially associate with fine suspended particles in the aqueous phase instead of the bottom sediments with coarse particles [4]. In addition, although SAs generally exhibit high solubility and hydrophilicity, several were also detected in sediments, and the number of species was comparable to that in surface water, this might be attributable to hydrogen bonding interactions between the polar groups of SAs and organic constituents in fine particles [35], causing only a small proportion of SAs to remain in sediments (0.03~0.96 ng/g).

Notably, the peak concentration distribution of antibiotics in sediments had significant temporal differences, mainly accumulating in the YRE in April and in the OYR in October.

### 3.4. Environmental Risk Assessment

**Figure 5:**
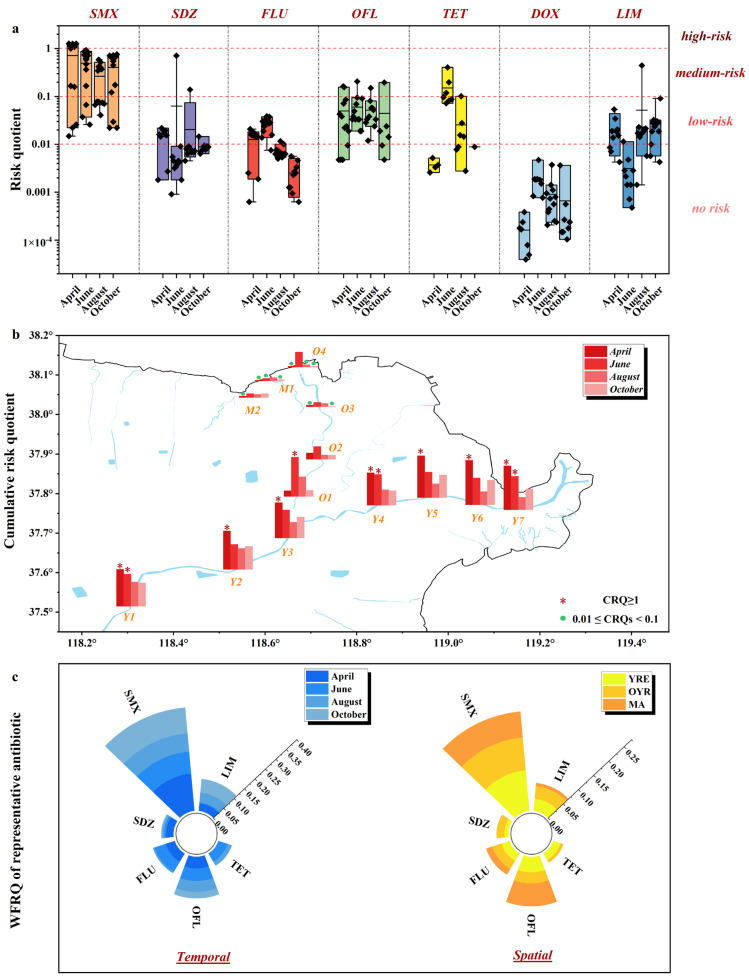
RQ (**a**), CRQ (**b**), and WFRQ (**c**) of representative antibiotics in surface water. Water samples were collected in April, June, August and October. The black square represented the RQ of a specific antibiotic at each sampling point. Map lines delineate study areas and do not necessarily depict accepted national boundaries.

RQs of antibiotics in surface water were calculated using available toxicity data (Table S3). The results showed that most of the individual representative antibiotics (e.g., SDZ, FLU, OFL, TET, DOX, and LIM) posed no or low risk to aquatic organisms, owing to the relatively high toxicity thresholds of these compounds. In particular, DOX exhibited negligible environmental risk in the YRD. In contrast, SMX presented medium or high risk in up to 84.6% of the sampling sites due to its high concentrations and low toxicity threshold. The maximum RQ of SMX reached 1.27 (Figure 5a), which is lower than that reported in the brackish water of German Bight (RQ = 6.6) [50], likely reflecting China’s tightened regulations on antibiotic prescription and usage in recent years.

Compared to individual antibiotics, multiple antibiotics coexisting in the environmental medium will cause greater toxicity due to the inevitable superposition effect. Therefore, the CRQs of representative antibiotics were calculated. As shown in Figure 5b, antibiotic pollution was mainly concentrated in the YRE, as reflected by CRQs in the sampling points in this area exceeding the high-ecological-risk red line (1.03–1.49) at least once in April and June. On the contrary, the ecological risk at sample points in the OYR was far lower (CRQ: 0.01~0.50), except at O1. In addition to the nearby hennery, the proximal connection with the mainstream river might contribute to the high risk of O1, as suggested by the higher CRQ during the water–sediment regulation period.

Notably, some predicted EC_50_ values might deviate from experimentally derived endpoints, because the ECOSAR software (v.2.2)operated based on an assumed baseline toxicity mechanism, which was unable to fully account for specific modes of action, mixture effects, or matrix interactions. This uncertainty could lead to either over- or underestimation of the derived PNEC values and subsequent RQ values. Therefore, the risk characterization of antibiotics based on predicted data should be interpreted with caution. Experimental toxicity tests in future studies could further reduce uncertainty.

Furthermore, positive correlations of high concentration–high concentration aggregation in YRE downstream and low concentration–low concentration aggregation in OYR downstream were observed in the spatial correlation data (Moran’s I = 0.373, z = 9.71, *p* = 0.001), which might be attributable to the contribution of SMX (Figure S6), as a similar pattern was found in previous reports [28]. More importantly, the need to prioritize control of SMX in the YRD was evidenced by its invariably largest share (37.70%~55.26%) of the total WFRQ value, regardless of spatial or temporal distribution (Figure 5c).

Compared with the surface water, sediment posed a more serious environmental risk. As previously reported, riverine antibiotics eventually accumulate in sediments due to hydrodynamic and wind–wave disturbances, which increase the residence time and the risk to aquatic ecosystems [51,52]. Although DOX has a relatively high toxicity threshold, its risk was still apparent, while other representative antibiotics were invariably high-risk. Without exception, the CRQs at all sample points reached the high-risk level, ranging from 1.70 to 306.73. The high RQs and CRQs in sediments could be explained by the equilibrium partitioning method used to derive PNEC_sed_ in this study. This method was based on three key assumptions (i.e., rapid and reversible exchange between sediment and porewater, bioavailability determined solely by porewater concentration, and similar sensitivity of benthic and pelagic organisms), which might introduce uncertainties [53], especially for ionizable antibiotics due to their more complex sorption mechanisms (e.g., hydrophobic interactions, double-layer reactions, ion exchange, ion polarization, hydrogen bonding). Moreover, partition coefficients are influenced by sediment particle composition, environmental pH, and redox potential. Therefore, imprecise partition coefficients or differences in species sensitivity could lead to significant over- or underestimation [54]. In this case, the Dutch Health Council has stated that the equilibrium partitioning method is applicable to organic, non-polar, and hydrophobic substances. Nonetheless, this method still has reference value for emerging pollutants lacking sediment toxicity data. Furthermore, according to the EU Technical Guidance Document, for the target antibiotics in sediment with logKow (−1.03~0.7) (Table S4), the potential underestimation of exposure was considered acceptable, and the criteria for triggering a sediment effect assessment were not met. Therefore, the use of the equilibrium partitioning method as a screening approach was scientifically justified, and our conclusion of high ecological risk can be considered a conservative estimate.

**Figure 6:**
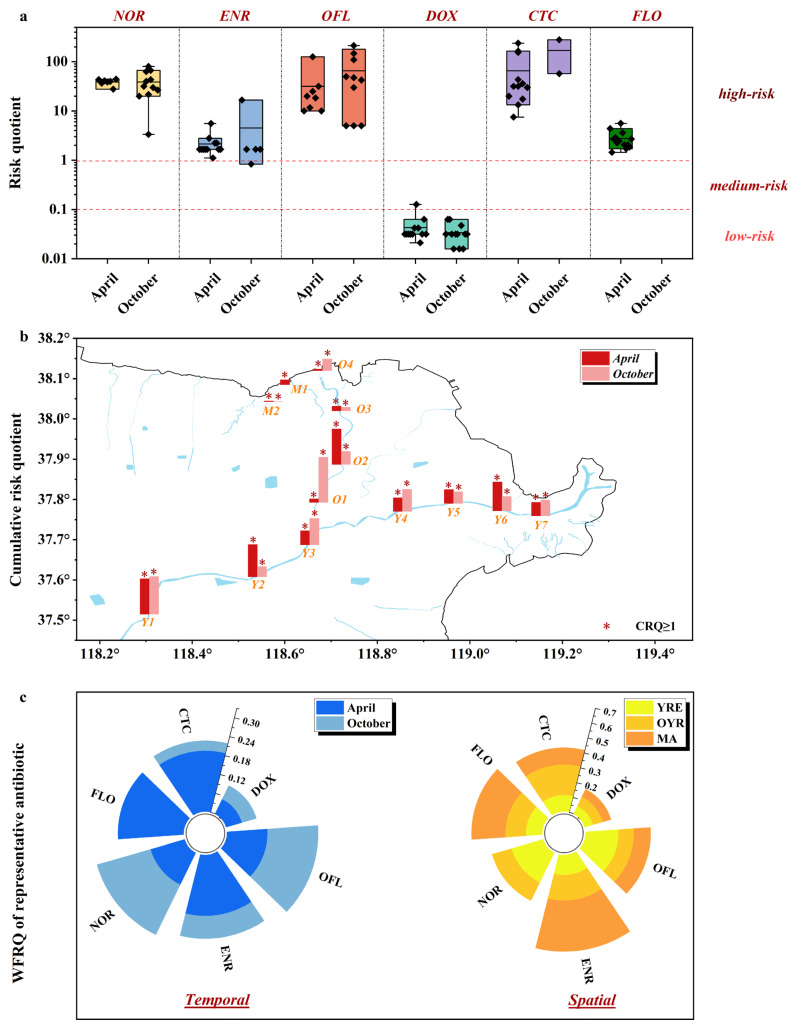
RQs (**a**), CRQs (**b**), and WFRQs (**c**) of representative antibiotics in sediment. Sediment samples were collected in April and October. No samples were collected in June or August. The black square represented the RQ of a specific antibiotic at each sampling point. Map lines delineate study areas and do not necessarily depict accepted national boundaries.

In this case, CRQs gradually decreased from upstream to downstream, and the offshore sampling points (O3, O4, and M1) showed low concentration–low concentration aggregation (Moran’s I = 0.132, z = 1.96, *p* = 0.042). The calculated results of temporal WFRQs indicate that the representative antibiotics in April were FLO, CTC, and ENR, which were replaced by NOR and OFL in October. Furthermore, the representative antibiotics showed significant spatial differences, with NOR and OFL dominating in the YRE, CTC, and ENR in the OYR, and ENR and FLO in the MA. The high temporal–spatial variability might be related to different consumption patterns and environmental behaviors. Notably, the risk posed by ENR, used in veterinary medicine, suggests the influence of livestock farming in the YRD (Figure 6 and Figure S7).

### 3.5. Source Apportionment of Representative Antibiotics

#### 3.5.1. Antibiotics in Surface Water

**Figure 7:**
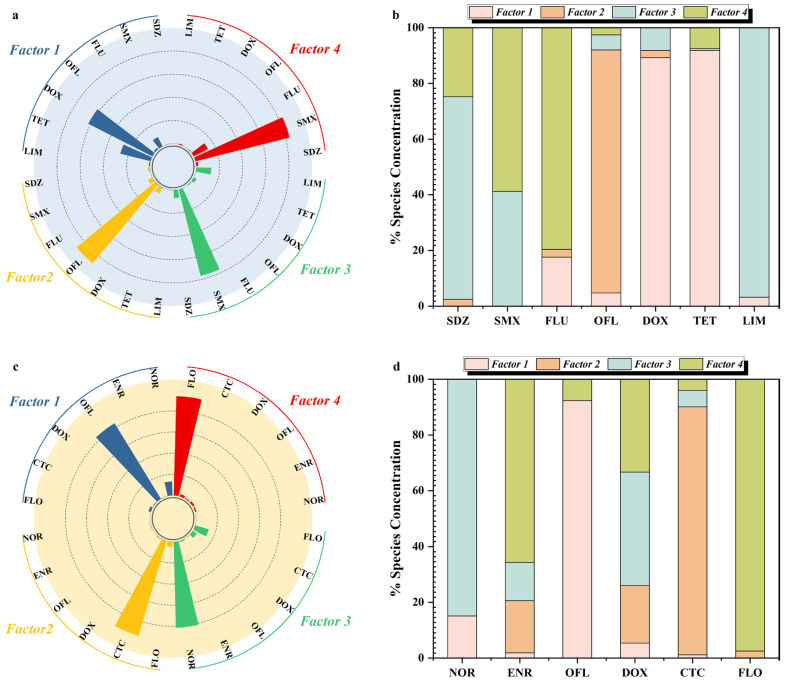
Source fingerprints and species apportionment of representative antibiotics in surface water (**a**,**b**) and sediment (**c**,**d**).

In surface water, four pollution factors with distinct chemical fingerprints were identified according to the percentage of the species sum and the percentage of the factor total in the PMF model (Figure 7a,b).

Specifically, Factor 1 was dominated by DOX (61.88%) and TET (26.81%), accounting for 88.69% of the factor total, with a simultaneous high percentage of the species sum (89.22%~91.83%). Additionally, the proportion of OFL (1.49%), which is banned for use in animals [2], can be ignored in the composition of Factor 1. This fingerprint feature is highly consistent with the medication pattern in the livestock and poultry farming industry, which usually employs TCs as growth promoters and prefers drugs for disease prevention [4,35]. Thus, Factor 1 likely originated from livestock and poultry farming sources, and its contribution appeared to be strongly restricted by hydrodynamic intensity conditions (Figure S8).

Furthermore, Factor 2 exhibited extremely strong specificity for OFL, which is widely used in clinical (human) medicine, as reflected by its high concentricity (88.17%) in the source fingerprint, suggesting that this factor might be attributable to anthropogenic sources. Further supporting this interpretation, Factor 2 contributes 87.30% of the OFL load in species apportionment. As previously reported, OFL is usually excreted in its original form or as active metabolites in urine and feces, ultimately entering the sewage treatment system. Therefore, this highly specific chemical fingerprint suggests that Factor 2 represents the input of human activities and medical wastewater. As shown in Figure S8, this contribution proportion had an insignificant variation range, consistent with the persistent emission of this typical sewage.

The fingerprint of Factor 3 was characterized by the combination of SMX (76.82%) and LIM (12.95%), with minor contributions from SDZ (6.83%). As is well documented, LIM and SDZ are common veterinary antibiotics, and their high contributions in the percentage of the species sum (96.79% and 72.70%, respectively) imply that Factor 3 might be associated with livestock and poultry farming activities [41,55]. However, SMX, which is used in both human and veterinary medicine, made up a considerable proportion of the fingerprint [56], indicating that the chemical fingerprint of Factor 3 was significantly different from that of Factor 1 (DOX/TET-dominated livestock and poultry farming sources); the prevalence of SMX might be explained by its high transport efficiency in runoff due to its high solubility. It is noteworthy that only 41.21% of SMX originated from Factor 3, pointing to diverse sources of this antibiotic. Considering the frequent application of organic fertilizer derived from livestock and poultry manure, as well as the large-scale flood irrigation pattern in farmland, Factor 3 could be interpreted as non-point source pollution caused by livestock-manure-derived agricultural runoff. As expected, the contribution sharply decreased in June, as the rising water level and extensive dilution of high discharge reduced the effect of the drug’s presence in agricultural runoff (Figure S8), which is highly consistent with the characteristics of agricultural non-point source pollution.

The fingerprint characteristics of Factor 4 were somewhat complex, consisting of a high proportion of SMX (83.50%) and FLU (13.70%). Meanwhile, species apportionment showed that 58.79% of SMX and 79.60% of FLU were contributed by Factor 4. As previously reported, FLU, as a QN, tends to adsorb on SPM and be gradually deposited into sediment due to its high partition coefficient (*K_d_*), extended half-life, and minimal degradation rate in rivers. Additionally, given its persistence and continuous input into surface water, SMX might absorb on SPM through hydrogen bonding. Therefore, these characteristics suggest that Factor 4 might be related to the release of SPM/sediment-adsorbed antibiotics. Furthermore, the uniform contribution along the route reflected the extensive mixing of the resuspended sediment. As illustrated in Figure S8, the relatively high contributions of Factor 4 in April and June significantly decreased in August and October, which might be explained by the depleted inventory after multiple water and sediment regulation operations during the flood season. Interestingly, the contribution of Factor 4 in June was similar to that in April, despite the significant difference in discharge between the two months (April: 276~460 vs. June: 904~1820 m^3^/s), because the standardized factor contributions of the PMF model output were relative data, not absolute fluxes.

#### 3.5.2. Antibiotics in Sediment

As shown in Figure 7c,d, the source fingerprint of Factor 1 consisted of 84.00% OFL and 13.10% NOR, and the species apportionment showed that 92.34% of OFL in sediment originated from this factor, suggesting typical human activity and medical wastewater sources. Sediments, as key sources and carriers of antibiotics, were extensively resuspended and redistributed under strong hydrodynamic conditions (June to September), resulting in an increased factor contribution at most reaches in October (Figure S9). In the YRE, the Y3–Y4 reaches showed transitions in the factor contribution, which might be attributable to the topographical change under sediment accumulation in recent years. Furthermore, the factor contribution in the Y4–Y7 reaches had inverse trends in April and October, which could be explained by the replacement of fine particles by coarse particles in the settled surface layer after multiple scouring events, which weakened the enrichment ability and shortened the transport path, despite similar low-flow conditions [39,57]. Additionally, the progressively decreased contribution along the OYR resulted from pulse inputs during the water–sediment regulation period; this intermittent and transitory connectivity of the river channels substantially restricted the long-distance transportation of sediment.

Differing from Factor 1, Factors 2 and 4 were both associated with the breeding industry, as reflected by the dominance of veterinary-specific antibiotics in the source fingerprint [28]. Factor 2 was mainly controlled by CTC (93.64%), with the corresponding contributions of CTC and DOX in species apportionment reaching 88.98% and 20.74%, respectively. Factor 4 was overwhelmingly controlled by FLO (94.02%) and simultaneously accounted for 97.52% of FLO and 65.68% of ENR in the sediment. In previous research, CTC and DOX were the most prevalent types of TCs found in animal manure (e.g., cattle, pigs, and poultry [4]), while FLO and ENR have been identified as representative antibiotics in aquaculture systems [58,59]. In addition, the relatively uniform distribution along the channel, without significant longitudinal gradients, agrees with the characteristics of non-point source antibiotic inputs driven by rainfall runoff, farmland drainage, or aquaculture water exchange. Therefore, Factors 2 and 4 could be tentatively attributed to livestock and poultry breeding sources and aquaculture sources, respectively.

Factor 3 was predominated by NOR (81.30%), used in both humans and animals, and included a significant proportion of veterinary CTC (13.36%), pointing to various sources (agricultural non-point source pollution, medical and domestic wastewater, etc.). More importantly, the temporal–spatial variations between this factor and Factor 4 in surface water (defined as the release of SPM/sediment-adsorbed antibiotics) are consistent, implying that this factor might represent a resuspended mixed phase formed by hydraulic disturbance of sediments.

## 4. Conclusions

This study revealed that the unique water–sediment regulation of the Yellow River fundamentally altered the typical seasonal antibiotic patterns observed in conventional river systems, with concentrations peaking in the regulation period rather than in dry seasons. The sharp surge in runoff during the water–sediment regulation period drove antibiotic transport while simultaneously remobilizing antibiotics in sediments through intense scouring. Ecological risk assessment identified SMX as the only priority control pollutant in surface water, while numerous high-risk antibiotics were present in sediment. More importantly, source apportionment identified hydrodynamic-driven sediment resuspension as a distinct internal cycling mechanism, distinguishing sediment-laden rivers from other aquatic systems. Consequently, antibiotic monitoring and early warning should be intensified during the water–sediment regulation period to capture peak risk windows in the YRD, focusing on SMX as a priority monitoring target in surface water and conducting regular risk assessment of antibiotics with high solid–water partition coefficients in sediment. Notably, this study did not assess antibiotic transformation products or their combined toxicity, and the risk quotients used might over- or underestimate actual impacts due to the lack of site-specific PNEC values, which should be further explored in future studies to improve the accuracy and specificity of risk control and management systems for antibiotics.

## Data Availability

The raw data supporting the conclusions of this article will be made available by the authors on request.

## Supplementary Materials

The following supporting information can be downloaded at: https://www.mdpi.com/article/10.3390/toxics14070552/s1. Figure S1: Schedule of sampling and the hydrological conditions of the YRD; Figure S2: Map of sampling points in the YRD; Figure S3: The schematic diagram of sediment extraction; Figure S4: Zoning analysis of dominant antibiotic; Figure S5: The average concentrations and detected frequencies of antibiotics in surface water; Figure S6: Spatial autocorrelation analysis of CRQ in surface water; Figure S7: Spatial autocorrelation analysis of CRQ in sediments; Figure S8: The spatiotemporal distribution of factors contribution in surface water; Figure S9: The spatiotemporal distribution of factors contribution in sediments; Table S1: Information of target antibiotics; Table S2: Method validation parameters for target antibiotics; Table S3: Toxicological data of representative antibiotics in surface water; Table S4: Toxicological data of representative antibiotics in sediments.
