# Supplementary material for: Occurrence, Ecological Risk, and Source Apportionment of Antibiotics in Surface Water and Sediment of Yellow River Delta
Source: Toxics. 2026 Jun 25;14(7):552. doi: 10.3390/toxics14070552 (PMC13418894; doi:10.3390/toxics14070552)
Supplement: Supplementary file 1 [file toxics-14-00552-s001.zip › toxics-4300246-supplementary.pdf]

Submission to ***Toxics***

Page: 18; Figure: 9; Table: 4

Supporting Information

Occurrence, Ecological Risk, and Source Apportionment of Antibiotics in Surface Water and Sediment of Yellow River Delta

Jinghao Wang, Shaohua Zhang, Yaoshen Fan, Feihe Kong, Renjie Huang, Shentang Dou*

Yellow River Institute of Hydraulic Research, Yellow River Conservancy Commission, Zhengzhou, 450003

Key Laboratory of Yellow River, Yellow River Estuary Eco-Hydrology Field Research Station, Ministry of Water Resources, Zhengzhou, 450003

***Corresponding Author：** E-mail: doushentang@126.com; Phone: +86 13838163560; Address: Yellow River Institute of Hydraulic Research, Zhengzhou, 450003, China

The following is included as additional supporting materials for this paper:

The detailed parameters used for PMF model performance are as follows:

- **Number of runs:** In total, 200 runs with random starts were performed; all 200 runs converged (100%).
- **Q values:** In source apportionment of surface water, the minimum robust Q (Q_{robust}) and true Q (Q_{true}) for the selected 4-factor solution were 1676.9 and 2145.6, respectively. In source apportionment of sediment, the minimum robust Q (Q_{robust}) and true Q (Q_{true}) for the selected 4-factor solution were 351.0 and 376.5, respectively.
- **Residuals:** In source apportionment of surface water, scaled residuals for the 4-factor solution showed that about 88% of all residuals fell within ±3. In source apportionment of sediment, scaled residuals for the 4-factor solution showed that about 82% of all residuals fell within ±3.
- **Uncertainty treatment:** The uncertainty treatment is described in Subsection 2.7. Data analysis. If concentration values (Con.) were below the method detection limit (MDL), the corresponding uncertainty (Unc.) was set as 5/6 MDL. Meanwhile, for Con. above MDL, the Unc. was calculated based on Eq. 2.9
- **Factor number selection:** This study evaluated 3- to 5-factor solutions and selected the 4-factor solution due to the presented Q_{robust} elbow, acceptable residuals, clear source separation, and good stability. The 3-factor solution showed underfitting, while the 5-factor solution showed overfitting.
- **Bootstrap/displacement analysis:** For the source apportionment of surface water, the mapping rates of bootstrap factors to the base run (Factors 1-4) were 100%, 82%, 100%, and 94%, respectively. DISP analysis showed zero factor swaps (swap counts = 0 for all factors).

In source apportionment of sediment, the mapping rates of bootstrap factors to the base run (Factors 1-4) were 90%, 86%, 97%, and 100%, respectively. DISP analysis showed zero factor swaps (swap counts = 0 for all factors).

- **Model stability:** Model stability was confirmed by 100% convergence across 200 runs, bootstrap mapping rates >80%, and zero factor swaps in DISP analysis.

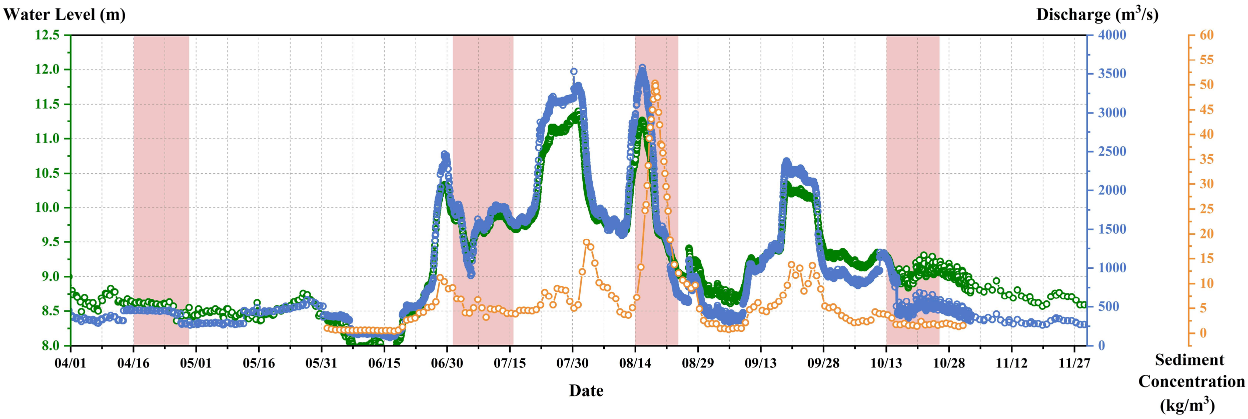

Fig. S1. Schedule of sampling and the hydrological conditions of the Yellow River estuary (red shading represents the sampling duration).

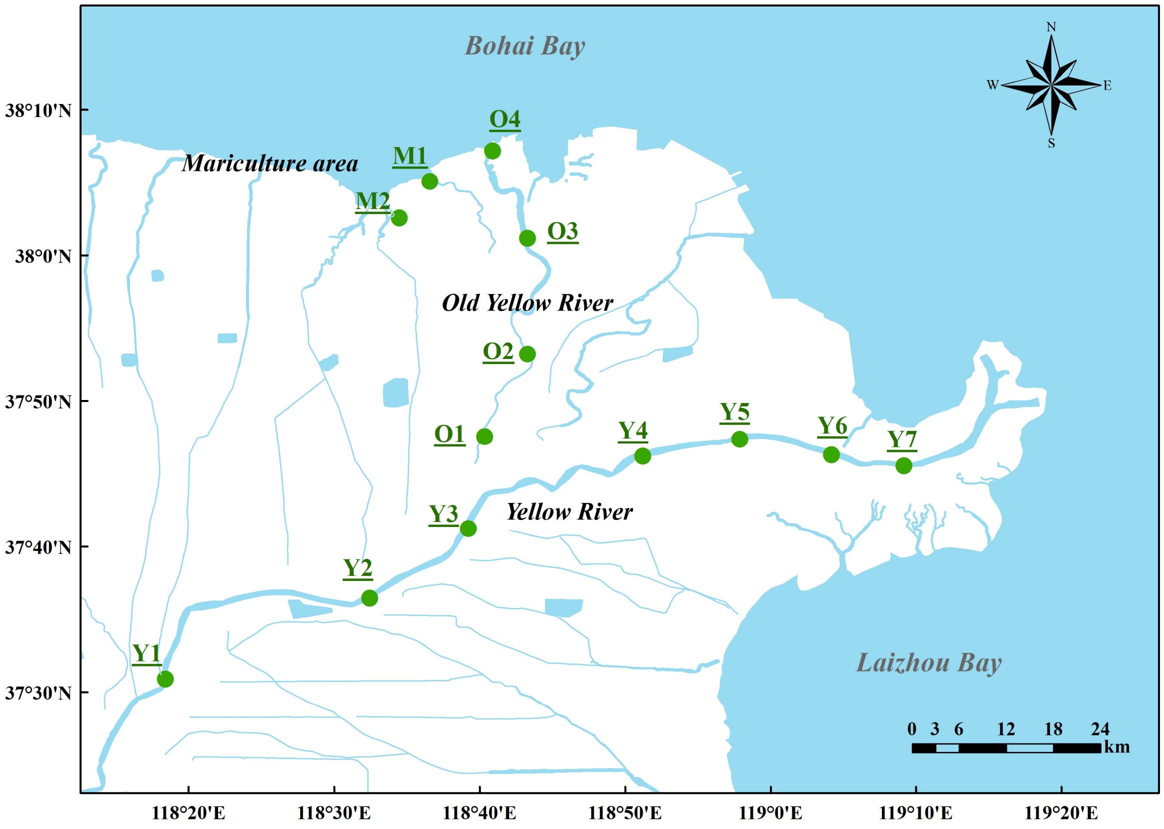

Fig. S2. Map of sampling points in the Yellow River Delta (map lines delineate study areas and do not necessarily depict accepted national boundaries).

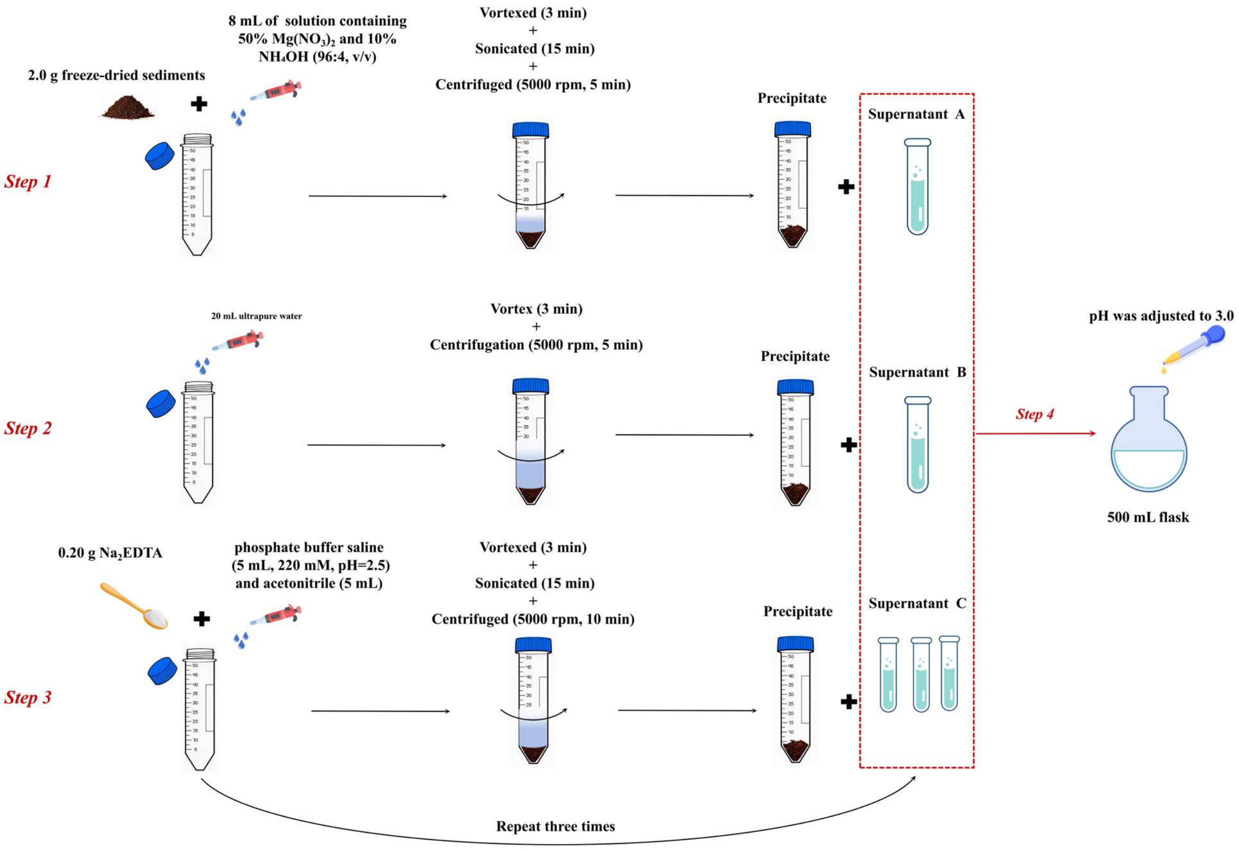

Fig. S3. The schematic diagram of sediment extraction.

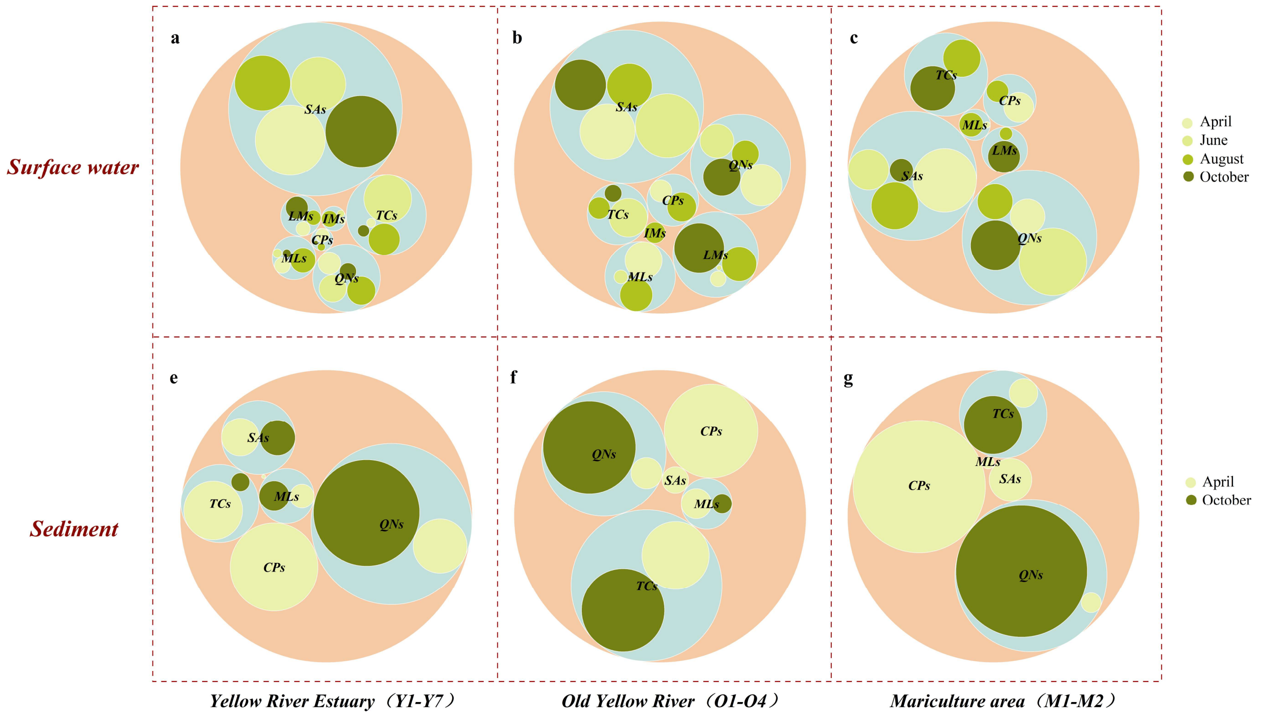

Fig. S4. Zoning analysis of dominant antibiotic (surface water: a-c, sediment: e-g).

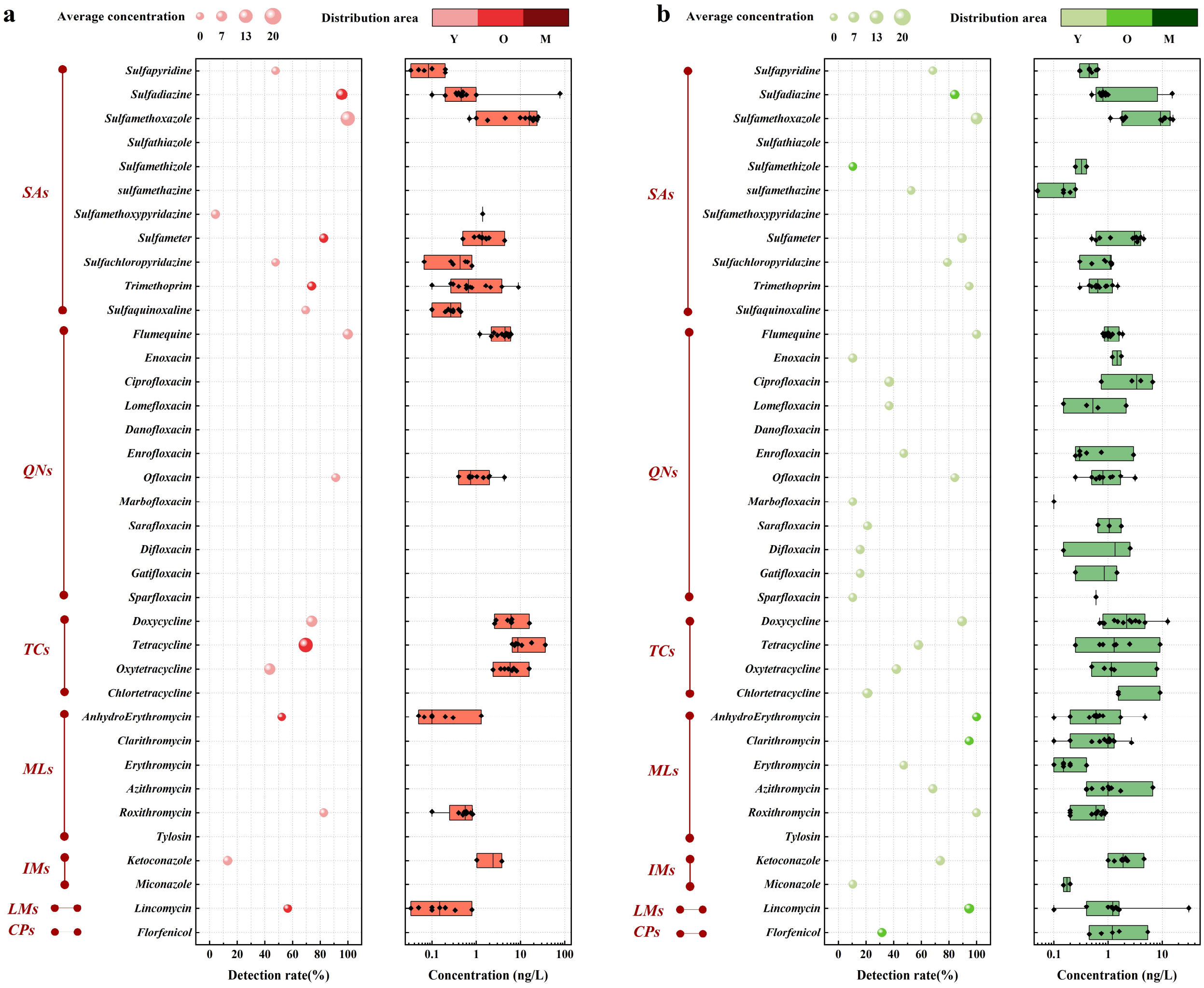

Fig. S5. The average concentrations and detected frequencies of antibiotics in surface water (a: June; b: August). Y, O, and M represent YRE, OYR, and MA, respectively.

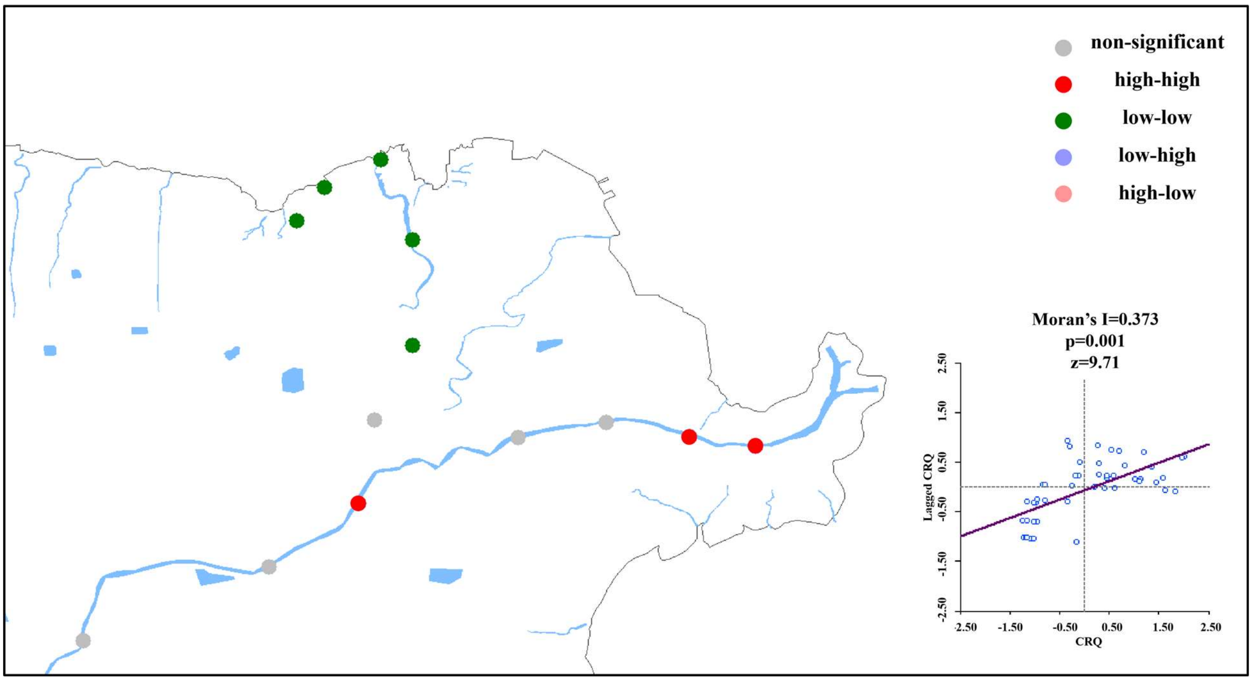

Fig. S6. Spatial autocorrelation analysis of CRQ in surface water (map lines delineate study areas and do not necessarily depict accepted national boundaries).

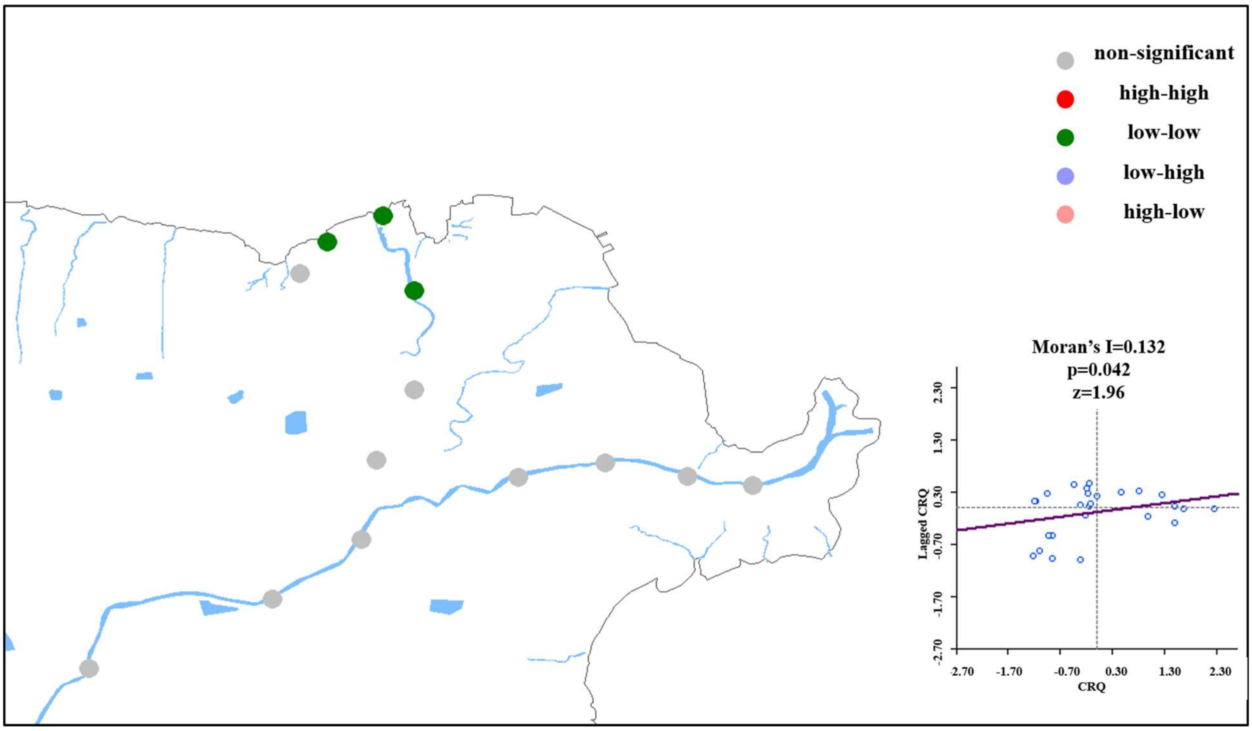

Fig. S7. Spatial autocorrelation analysis of CRQ in sediments (map lines delineate study areas and do not necessarily depict accepted national boundaries).

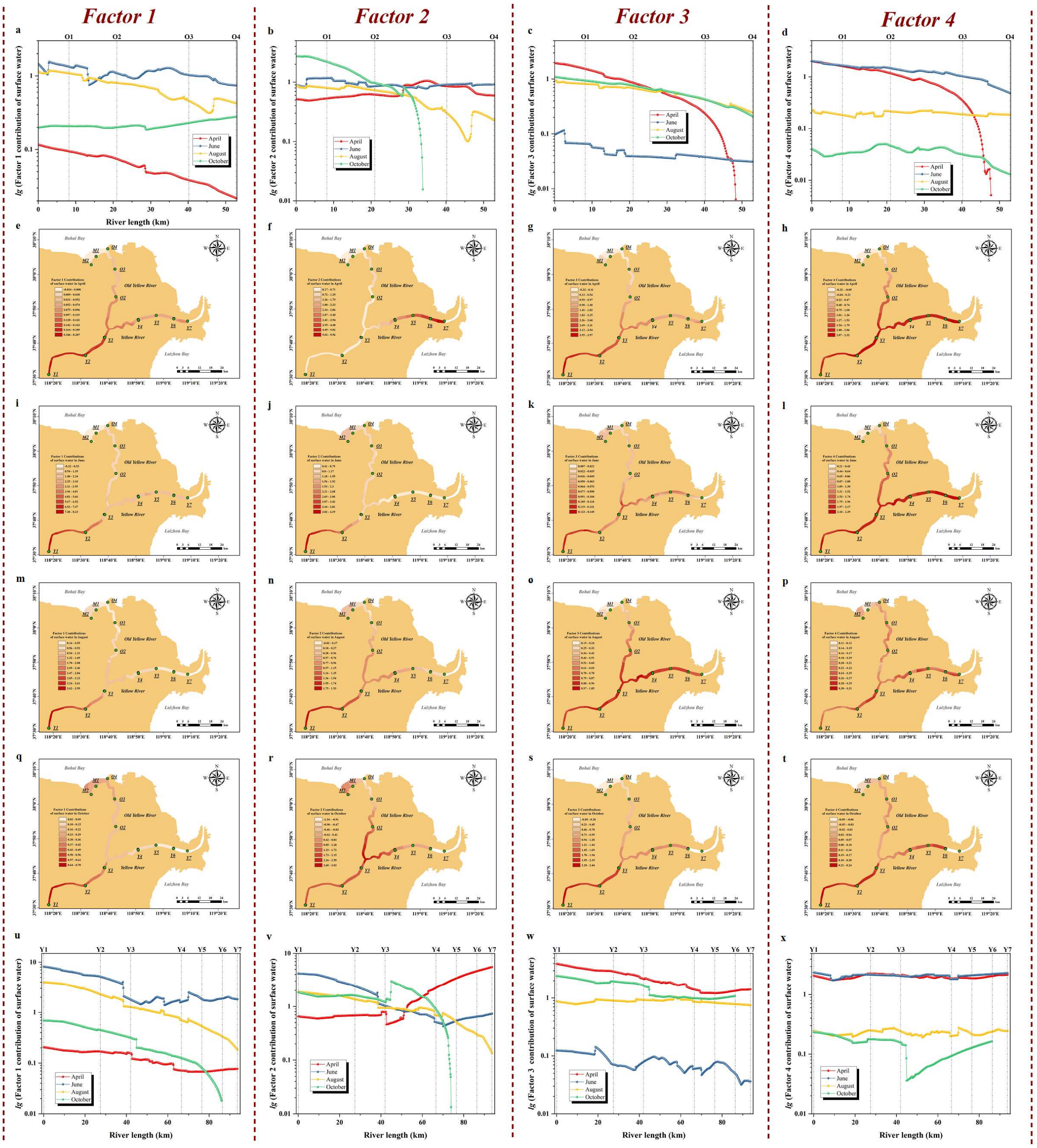

Fig. S8. The spatiotemporal distribution of factors' contribution in surface water (a-d: the section line of factor contribution in OYE; u-x: the section line of factor contribution in YRE; e-h: the spatial distribution of factor in April; i-l: the spatial distribution of factor in June; m-p: the spatial distribution of factor in August; q-t: the spatial distribution of factor in October; map lines delineate study areas and do not necessarily depict accepted national boundaries).

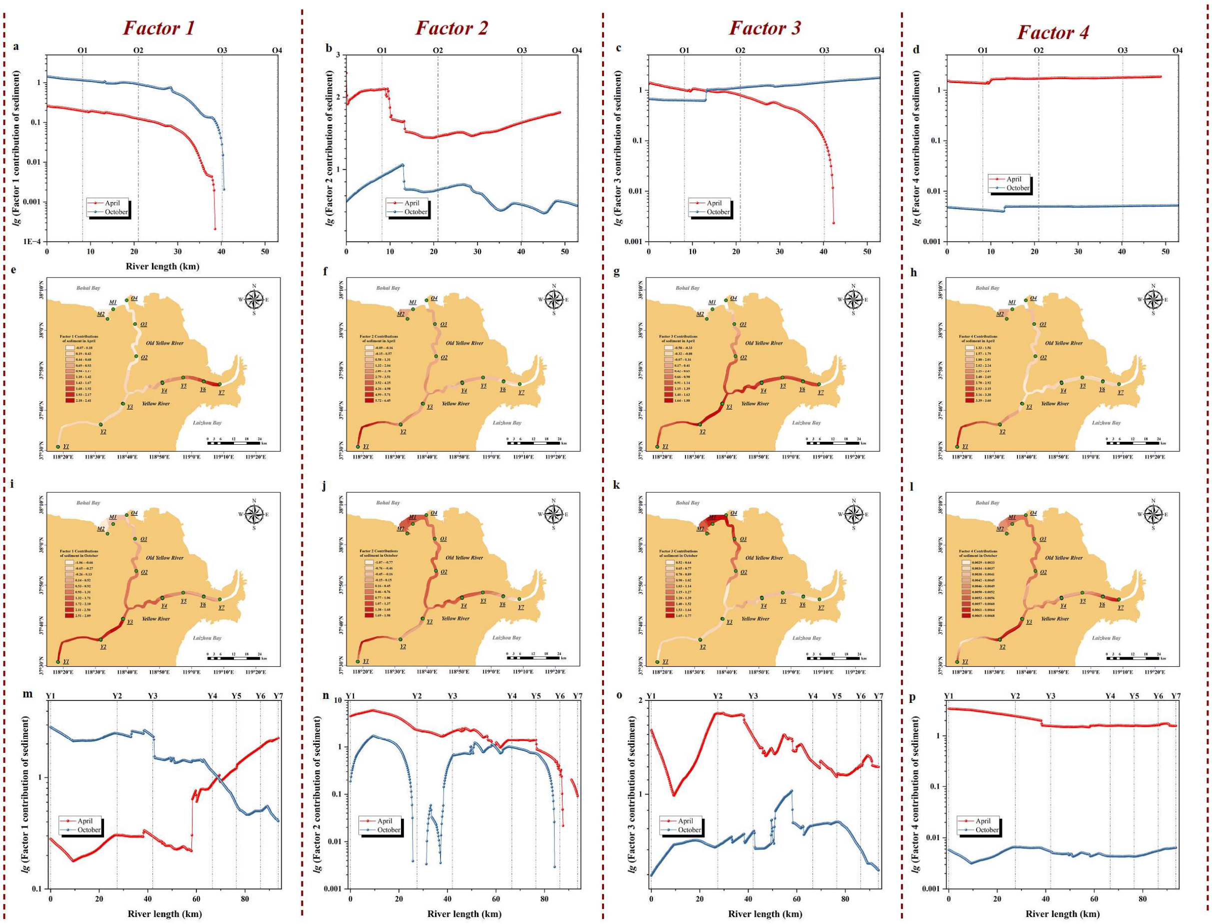

Fig. S9. The spatiotemporal distribution of factors' contribution in sediment (a-d: the section line of factor contribution in OYE; m-p: the section line of factor contribution in YRE; e-h: the spatial distribution of factor in April; i-l: the spatial distribution of factor in October; map lines delineate study areas and do not necessarily depict accepted national boundaries).

Table S1. Information on target antibiotics.

Class	Compounds	Relative molecular mass	Molecular formula
Sulfonamides （SAs）	Sulfacetamide	214.24	$C_8H_{10}N_2SO_3$
	Sulfapyridine	249.29	$C_{11}H_{11}N_3O_2S$
	Sulfadiazine	250.28	$C_{10}H_{10}N_4O_2S$
	Sulfamethoxazole	253.28	$C_{10}H_{11}N_3O_3S$
	Sulfathiazole	255.32	$C_9H_9N_3O_2S_2$
	Sulfamerazine	264.30	$C_{11}H_{12}N_4O_2S$
	Sulfisoxazole	267.30	$C_{11}H_{13}N_3O_3S$
	Sulfamethizole	270.33	$C_9H_{10}N_4O_2S_2$
	Sulfamethazine	278.33	$C_{12}H_{14}N_4O_2S$
	Sulfamonomethoxine	280.30	$C_{11}H_{12}N_4O_3S$
	Sulfamethoxypyridazine	280.30	$C_{11}H_{12}N_4O_3S$
	Sulfameter	280.30	$C_{11}H_{12}N_4O_3S$
	Sulfachloropyridazine	284.72	$C_{10}H_9ClN_4O_2S$
	Sulfaquinoxaline	300.34	$C_{14}H_{12}N_4O_2S$
	Sulfadoxine	310.33	$C_{12}H_{14}N_4O_4S$
	Sulfadimethoxine	310.33	$C_{12}H_{14}N_4O_4S$
	Sulfaphenazole	314.36	$C_{15}H_{14}N_4O_2S$
	Trimethoprim	290.32	$C_{14}H_{18}N_4O_3$
Quinolones （QNs）	Norfloxacin	319.33	$C_{16}H_{18}FN_3O_3$
	Enoxacin	320.32	$C_{15}H_{17}FN_4O_3$
	Ciprofloxacin	331.34	$C_{17}H_{18}FN_3O_3$
	Pefloxacin	333.36	$C_{17}H_{20}FN_3O_3$
	Lomefloxacin	351.35	$C_{17}H_{19}F_2N_3O_3$
	Danofloxacin	357.38	$C_{19}H_{20}FN_3O_3$
	Enrofloxacin	359.40	$C_{19}H_{22}FN_3O_3$
	Ofloxacin	361.37	$C_{18}H_{20}FN_3O_4$
	Marbofloxacin	362.36	$C_{17}H_{19}FN_4O_4$
	Fleroxacin	369.34	$C_{17}H_{18}F_3N_3O_3$
	Sarafloxacin	385.36	$C_{20}H_{17}F_2N_3O_3$
	Difloxacin	399.39	$C_{21}H_{19}F_2N_3O_3$
	Gatifloxacin	375.39	$C_{19}H_{22}FN_3O_4$
	Sparfloxacin	392.40	$C_{19}H_{22}F_2N_4O_3$
	Oxolinic acid	261.23	$C_{13}H_{11}NO_5$
	Flumequine	261.25	$C_{14}H_{12}FNO_3$

Macrolides（MLs）	Clarithromycin	747.95	$C_{38}H_{69}NO_{13}$
	Erythromycin	733.94	$C_{37}H_{67}NO_{13}$
	Anhydroerythromycin	715.91	$C_{37}H_{65}NO_{12}$
	Azithromycin	749.00	$C_{38}H_{72}N_2O_{12}$
	Roxithromycin	837.05	$C_{41}H_{76}N_2O_{15}$
	Tylosin	916.12	$C_{46}H_{77}NO_{17}$
Tetracyclines（TCs）	Tetracycline	444.45	$C_{22}H_{24}N_2O_8$
	Oxytetracycline	460.43	$C_{22}H_{24}N_2O_9$
	Chlortetracycline	478.88	$C_{22}H_{23}ClN_2O_8$
	Doxycycline	444.44	$C_{22}H_{24}N_2O_8$
chloramphenicols（CPs）	Thiamphenicol	356.22	$C_{12}H_{15}Cl_2NO_5S$
	Chloramphenicol	323.13	$C_{11}H_{12}Cl_2N_2O_5$
	Florfenicol	358.21	$C_{12}H_{14}Cl_2FNO_4S$
Lincomycins（LMs）	Lincomycin	406.54	$C_{18}H_{34}N_2O_6S$
imidazoles（IMs）	Ketoconazole	531.43	$C_{26}H_{28}Cl_2N_4O_4$
	Miconazole	416.13	$C_{18}H_{14}Cl_4N_2O$

Table S2 Method validation parameters for target antibiotics.

Compounds	LOD	LOQ	Recovery (%)	RSD (n=6)	Recovery (%)	RSD (n=6)	Linearity ranges	Correlation coefficients	R^2	Matrix effects (%)	
										Water	Sediment
Sulfacetamide	0.09	0.30	83.7	5.1	78.2	8.8	1-200ug/L	y = 935.9x+34.2	0.9993	133.74	126.92
Sulfapyridine	0.13	0.42	89.0	11.2	86.3	9.4	1-200ug/L	y = 746.2x-35.5	0.9999	106.04	109.36
Sulfadiazine	0.06	0.20	108.4	6.7	89.6	8.1	1-200ug/L	y = 735.2x-30.8	0.9996	117.08	118.52
Sulfamethoxazole	0.08	0.27	82.3	4.6	86.9	4.2	1-200ug/L	y = 670.9x+10.8	0.9997	101.96	104.4
Sulfathiazole	0.04	0.13	77.6	8.8	96.5	10.5	1-200ug/L	y = 591.9x - 38.0	0.9999	112.72	112.24
Oxolinic acid	0.04	0.14	84.9	5	96.3	4.1	1-200ug/L	y = 5406.4x - 368.1	0.9997	104.66	95.22
Flumequine	0.06	0.20	78.2	1.5	98.7	4.8	1-200ug/L	y = 1282.1x + 28.8	0.9998	87.58	72.04
Sulfamerazine	0.04	0.15	90.8	6.8	106.1	10.4	1-200ug/L	y = 642.3x - 63.3	0.9997	107.58	113.16
Sulfisoxazole	0.05	0.16	80.9	3.7	80.6	6.6	1-200ug/L	y = 1043.9x - 119.6	0.9998	101.24	101.6
Sulfamethizole	0.05	0.15	81.0	8.6	93.5	7.9	1-200ug/L	y = 661.7x + 19.5	0.9998	101.48	109.3
Sulfamethazine	0.01	0.05	83.8	6.5	94.6	7.5	1-200ug/L	y = 918.0x - 36.7	0.9999	105.56	127.26
Sulfamonomethoxine	0.04	0.12	78.2	7.5	93.9	7.1	1-200ug/L	y = 303.5x - 45.7	0.9998	107.72	112.14
Sulfamethoxypyridazine	0.14	0.46	91.4	10	89.3	9.6	1-200ug/L	y = 57.4x - 0.1	0.9982	114.04	88.12
Sulfameter	0.05	0.16	88.2	7.8	99.2	6	1-200ug/L	y = 399.3x - 54.5	0.9997	110.86	114.3
Sulfachloropyridazine	0.06	0.21	79.4	6.3	89.8	12.1	1-200ug/L	y = 430.6x - 36.2	0.9998	97.88	107.9
Trimethoprim	0.02	0.06	80.0	7.3	95.2	5.1	1-200ug/L	y = 1403.0x - 8.8	0.9994	102.94	98.46
Sulfaquinoxaline	0.08	0.26	79.3	3.9	105.6	7.9	1-200ug/L	y = 733.9x - 47.5	0.9998	104.04	103.5
Sulfadoxine	0.03	0.12	80.1	8	95.0	6.1	1-200ug/L	y = 1627.7x - 31.1	0.9999	101.3	108
Sulfadimethoxine	0.02	0.07	82.9	7.7	99.6	4.6	1-200ug/L	y = 1047.2x - 102.9	0.9999	105.64	106.44
Sulfaphenazole	0.02	0.08	80.0	4.8	98.8	5	1-200ug/L	y = 390.7x - 18.8	0.9999	102.14	98.74
Norfloxacin	0.08	0.26	81.4	4.2	94.1	8.7	1-200ug/L	y = 413.3x - 59.9	0.9997	99.72	93.7
Enoxacin	0.10	0.33	79.0	8.2	86.8	10.9	1-200ug/L	y = 482.3x - 36.2	0.9994	98.98	91.18
Ciprofloxacin	0.06	0.18	86.4	5	99.1	6	1-200ug/L	y = 735.6x - 104.3	0.9997	96.42	84.46

Pefloxacin	0.05	0.17	90.1	8.8	87.7	8.8	1-200ug/L	y = 1592.3x - 40.5	0.9992	104.92	93.72
Lomefloxacin	0.02	0.07	87.6	6.7	79.1	6.5	1-200ug/L	y = 1114.5x - 17.0	0.9991	102.16	91.16
Danofloxacin	0.05	0.18	73.3	3	84.5	5.6	1-200ug/L	y = 1278.8x - 84.3	0.9994	102.04	93.44
Enrofloxacin	0.04	0.14	78.2	7.5	81.6	5.6	1-200ug/L	y = 830.0x - 35.8	0.9996	105.08	98.86
Ofloxacin	0.02	0.08	79.2	4.1	83.8	5.9	1-200ug/L	y = 1233.0x + 90.0	0.9990	98.6	88.88
Marbofloxacin	0.12	0.40	80.6	4.1	91.1	11.5	1-200ug/L	y = 3665.5x - 238.7	0.9998	100.06	93.56
Fleroxacin	0.07	0.23	79.2	7.8	89.4	6.6	1-200ug/L	y = 1266.3x - 147.9	0.9997	103.62	94.4
Gatifloxacin	0.04	0.13	72.7	4	82.4	6.8	1-200ug/L	y = 538.7x - 27.6	1.0000	102.34	92.28
Sarafloxacin	0.05	0.18	75.1	5.8	84.6	6.1	1-200ug/L	y = 360.2x - 10.9	0.9998	103.04	94.72
Sparfloxacin	0.01	0.04	77.9	3.7	86.0	10	1-200ug/L	y = 911.8x - 48.6	0.9999	101.44	93.28
Difloxacin	0.05	0.18	76.1	3	94.0	8.8	1-200ug/L	y = 380.5x + 28.4	0.9990	105.72	97.16
Lincomycin	0.02	0.06	65.3	14.1	60.8	14.3	1-200ug/L	y = 2017.6x + 55.2	0.9992	107.42	108.72
Miconazole	0.01	0.03	83.5	5.3	84.6	9.6	1-200ug/L	y = 120.0x - 19.0	0.9993	88.86	66.32
Doxycycline	0.04	0.14	100.2	0.8	97.8	3.7	1-200ug/L	y = 430.0x - 90.3	0.9992	98.48	104.6
Tetracycline	0.08	0.27	97.2	5.9	95.9	10.4	1-200ug/L	y = 121.5x - 44.1	0.9992	105.26	110.72
Oxytetracycline	0.09	0.29	105.1	3.4	95.9	8.2	1-200ug/L	y = 146.7x + 58.7	0.9991	103.02	109.86
Chlortetracycline	0.16	0.52	105.5	6.1	98.5	9.5	1-200ug/L	y = 125.8x - 31.8	0.9991	119.82	106.8
Ketoconazole	0.25	0.78	79.8	12.2	72.5	6.8	1-200ug/L	y = 145.6x - 49.8	0.9993	82.46	62.9
AnhydroErythromycin	0.01	0.03	85.8	4	92.2	7.5	1-200ug/L	y = 3242.2x + 58.7	0.9994	82.3	63.98
Erythromycin	0.07	0.24	77.9	7.8	76.1	4.2	1-200ug/L	y = 3540.7x - 367.1	0.9993	120.98	97.52
Clarithromycin	0.01	0.03	109.6	3.1	98.1	7.1	1-200ug/L	y = 8068.6x - 483.9	0.9999	92.72	71.64
Azithromycin	0.01	0.04	79.3	6.1	99.6	5.5	1-200ug/L	y = 2101.1x - 66.1	0.9999	91.36	70.38
Roxithromycin	0.02	0.05	75.6	5.3	91.3	13.2	1-200ug/L	y = 2831.4x - 368.3	0.9995	104.02	91.54
Tylosin	0.03	0.09	67.3	11.2	82.3	6	1-200ug/L	y = 123.8x - 14.1	0.9995	94.1	80.68
Chloramphenicol	0.05	0.16	93.9	3.9	90.0	5.7	1-200ug/L	y = 235.5x + 18.4	0.9998	102.68	99.78
Thiamphenicol	0.09	0.29	90.1	4.6	100.0	8.2	1-200ug/L	y = 185.1x + 35.9	0.9991	100.56	88.9
Florfenicol	0.07	0.25	74.9	7.5	101.9	5.3	1-200ug/L	y = 88.4x -19.7	0.9995	94.56	78.32

Table S3. Toxicological data of representative antibiotics in surface water.

Antibiotic	Class	EC_{50}（mg/L）	$PNEC_{water}$（ng/L）
	Algae	0.027	
SMX	Crustacean	0.21	27
	Fish	27.35	
	Algae	0.11	
SDZ	Crustacean	57.00	110
	Fish	907*	
	Algae	0.159	
FLU	Crustacean	59	159
	Fish	510*	
	Algae	0.021	
OFL	Crustacean	3.13	21
	Fish	19400*	
	Algae	0.09	
TET	Crustacean	10	90
	Fish	260.5	
	Algae	3.37*	
DOX	Crustacean	2000*	3370
	Fish	820*	
	Algae	0.07	
LIM	Crustacean	7.2	70
	Fish	1040*	

Data was obtained from the Ecotoxicology Database (ECOTOX) of the United States or predicted using ECOSAR software (marked with *).

Table S4. Toxicological data of representative antibiotics in sediment.

Antibiotic	K_{OC} (L/kg)	$logK_{OW}$	Class	EC_{50} (mg/L)	$PNEC_{water}$ (ng/L)	$PNEC_{sed}$ (μg/kg)
			Algae	0.038		
NOR	1.70	-1.03	Crustacean	0.88	38	0.03
			Fish	20100*		
			Algae	0.049		
ENR	20.38	0.70	Crustacean	14.3	49	0.06
			Fish	100		
			Algae	0.021		
OFL	4.27	-0.39	Crustacean	3.13	21	0.02
			Fish	19400*		
			Algae	3.37*		
DOX	7.25	-0.02	Crustacean	2000*	3370	3.17
			Fish	820*		
			Algae	0.05		
CTC	3.07	-0.62	Crustacean	8.20	50	0.04
			Fish	78.9		
			Algae	2.3		
FLO	7.05	-0.04	Crustacean	64	2300	2.15
			Fish	/		

Data was obtained from the Ecotoxicology Database (ECOTOX) of the United States or predicted using ECOSAR software (marked with *).
